# Crisis Communication Saved Lives in Israel

**DOI:** 10.3389/fpubh.2014.00222

**Published:** 2014-11-04

**Authors:** Gilead Shenhar

**Affiliations:** ^1^Gertner Institute, Ramat Gan, Israel; ^2^Disaster Management, Tel Aviv University, Tel Aviv, Israel

**Keywords:** crisis communication, crisis management, Israel, operation protective edge, Iron Dome

Israel launched “Operation Protective Edge” (1, 2) against Hamas, Islamic Jihad, and other terrorist groups in Gaza during August–September 2014 to restore safety and security to its citizens; the operation had the limited objective of ending the continuous rocket and mortars firings and to destroy the tunnels that were dug under Israeli sovereign territory for the purpose of terror killing and kidnapping. Israelis in communities close to Gaza had become targets of intermittent shelling from Gaza for the past fourteen years. Living under fire has become a routine reality for Israeli residents causing, significant psychological trauma and anxiety, which is the intention of terrorist activity. During the operation, more than 4382 rockets were launched over Israel and more than 70% Israelis live within range of Hamas’ rockets (2).

In response, Israel has developed a two-layered defensive strategy to address this ongoing situation: first, an active-defense system with the “Iron Dome” designed to intercept and destroy rockets that set to land in populated areas; second, a passive-defense system based on air alerts sirens, Urban Search And Rescue teams, medical system including hospitals, Emergency Medical Service, influencing and directing public behavior through crisis communication. While many people were aware that “Iron Dome” intercepted most of the incoming rockets that would have caused many Israeli casualties, few may be aware of the second strategy that has spawned a sophisticated civil defense culture. That may be constructive in understanding how Israel emerged from the conflict with relatively few civilian casualties and how other countries could adapt this system if ever under sustained terror attack.

The aim of crisis communication is to enable the public to be knowledgeable concerning recommended behavior during an attack and minimize the disruption of normal life. Considering the inability of “Iron Dome” system to intercept 100% of the rockets aimed at the civilian population, crisis communication is an important component of this second layer of civil defense. The Iron Dome system intercepted 692 (2) of the rockets, whose trajectory would land in populated areas, resulting in a high rate of successful interceptions (90%). Because of the two-tiered method, the loss of human life was very limited. Five civilians were killed and 684 Israeli civilians were injured inflicted mostly by light injuries.

The Israeli Civil Protection (Home front Command) educated the Israeli public concerning the techniques and procedures they should implement during a rocket attack; individuals were required to enter the best available protected spaces depending upon their location. The time given ranged from 15 s in areas near the Gaza Strip, and up to 90 s in areas more than 40 km from the Gaza Strip. These guidelines, in conjunction with the full cooperation of the civilian population, saved a significant number of lives, reducing casualties as well as the volume of casualties needing medical assistance. Research on crisis communication has demonstrated that pre-emptive knowledge has a dramatic impact on the public’s designated behavior to protect themselves during crises and reduces anxiety (3, 4).

The crisis communication platform was achieved by activating a pre-planned system immediately upon the onset of this operation. The system consisted of multiple components including call centers (which received more than 330,000 calls during the first 45 days of operation, with a high load of 40,000 calls during 1 day) (5); guidelines in newspapers; websites (in several languages with more than 1.65 million enterers), and social media (Facebook and Twitter). The new social media help to deliver Civil Protection messages to the public; however, some individuals spread rumors, mostly through WhatsApp. Therefore, there was a need to monitor the new media and to refute rumors as fast as possible. The Civil Protection prepared short clips that provided messages, instructions, and information regarding vital services. They also broadcasted in various languages, constantly transmitting on YouTube. Special programs were prepared for children using well-known characters to disseminate child-friendly messages.

National spokespersons were nominated, and each assigned to the main television and radio news channels. These spokespersons were the Civil Protection representatives in these channels; they spoke uniformly in clear, simple messaging that provided life-saving information, and supported the public by calming them. The advanced training and coordination of the three main spokespersons in the media channels facilitated a quick and smooth process throughout the operation. The spokespersons participating most of the media programs in a 24/7 span time frame, during the days of the operation. They became known faces to the public. During the operation, there was a significant increase in the volume of public listening and network viewing, allowing the public to be more exposed to the Civil Protection messages.

The increased attentiveness benefited citizen’s better understanding of the information guidelines, as well as to improve proper responses during rocket attacks (5). Quickly entering their protected spaces protected the public; even when the building they stayed in was hit, causing heavy destruction, people survived. Survivors were used as important examples, assisting in persuading the public to act according to instructions.

The Prime Minister, media reporters, and local leadership assisted in emphasizing the importance of abiding by the Civil Protection, saving many lives. They were convinced of the efficacy of crisis communications, after witnessing many occasions where civilians were saved as a result of acting according to Civil Protection guidelines.

In summary, the Civil Protection executed, throughout the operation, a crisis communication plan designed to provide with the public guidelines, tools, knowledge, and skills in order to protect civilians. The guidance given to the population while ensuring the principle of simplicity, clarity, continuity, reliability, transparency, and uniformity of the messages led the public to strictly abide. We believe that this had an important effect on reduction of the volume of casualties and saving lives.

Israel’s Civil Protection should become prepared for larger scaled events when we might absorb larger volumes of missiles and rockets. This future risk creates major challenges for crisis communication systems. As it is believed that future challenges, resulting from massive rocket or terror tunnel attacks are expected, it is crucial to prepare and strengthen the crisis communication system within the second layer of defense, in order to effectively meet these challenges.

## Conflict of Interest Statement

The author declares that the research was conducted in the absence of any commercial or financial relationships that could be construed as a potential conflict of interest.
